# Deformation Failure Characteristics of Coal Body and Mining Induced Stress Evolution Law

**DOI:** 10.1155/2014/714507

**Published:** 2014-05-19

**Authors:** Zhijie Wen, Guanglong Qu, Jinhao Wen, Yongkui Shi, Chuanyang Jia

**Affiliations:** ^1^State Key Laboratory of Mining Disaster Prevention and Control, Shandong University of Science and Technology, Qingdao, Shandong 266590, China; ^2^Research Center of Geotechnical and Structural Engineering, Shandong University, Jinan, Shandong 250061, China; ^3^Aalto University, 00076 Aalto, Finland

## Abstract

The results of the interaction between coal failure and mining pressure field evolution during mining are presented. Not only the mechanical model of stope and its relative structure division, but also the failure and behavior characteristic of coal body under different mining stages are built and demonstrated. Namely, the breaking arch and stress arch which influence the mining area are quantified calculated. A systematic method of stress field distribution is worked out. All this indicates that the pore distribution of coal body with different compressed volume has fractal character; it appears to be the linear relationship between propagation range of internal stress field and compressed volume of coal body and nonlinear relationship between the range of outburst coal mass and the number of pores which is influenced by mining pressure. The results provide theory reference for the research on the range of mining-induced stress and broken coal wall.

## 1. Introduction

With the deterioration of occurrence condition of coal resources, mechanical environment for coal mining, organization structure of coal and rock mass, its mechanical behavior and failure characteristics become complicated, so the characteristics of engineering response change [1–3]. Meanwhile, the large space mining model complicates time-space relationship and kinetics features of mining stress field rock burst and coal and gas outburst, which causes serious damage and a number of casualties. The main reasons for these problems are lack of knowledge of the space-time evolution law of mining stress field with different mining conditions, roadway excavation and maintenance, and the advance of heading face at the wrong time and space.

The domestic and foreign scholars usually use classical mechanics, (e.g., elastic-plastic mechanics) to study the damage development process of coal body and the mining stress field that loads on the coal body during the process under the condition of mining activity [4, 5]. In this analytical method, ideal coal body is under the condition of static stress. But coal body in the stope is under dynamic loading induced by the bending and breaking of the overlaying strata, which weakens the mechanical properties of coal body and continuously transfer the mining stress inside the coal body [6]. So the quantitative study on the space-time evolution mechanism and its law of mining stress field with the deterioration development of mechanical properties of coal body is necessary [7]. It is helpful to the roadway excavation, maintenance, and the advance of the heading face, which has very important practical significance.

## 2. Macromechanical Structure of the Stope

The overlying strata of coal seam can be divided into two parts: the outrange of overlying strata spatial structure of stope that consists of no significant move rock beams in the stress arch that does not move significantly and the overlying strata spatial structure that is composed of kinetic rock strata in the breaking arch that affects the ground pressure of stope directly.

**Figure 1:**
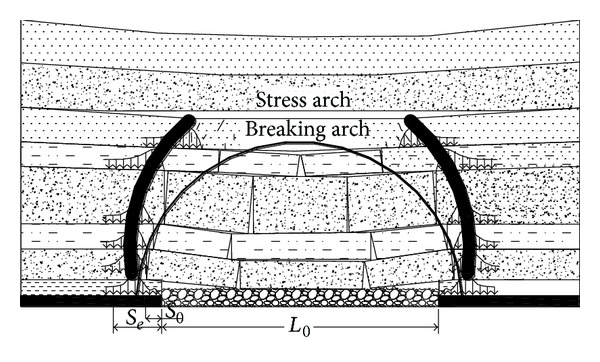
The “two-arch” structure of mining stope.

With the advance of heading face, the hanging space of stope continuously increases, to a certain degree, the overlying strata start to break from bottom to top, which forms the breaking arch [8–10]. At the same time, the stress in the spatial structure strata is redistributed and the gravity of overlying strata, originally supported by the mining coal body, is loaded on the two sides of the coal body and rock mass at the same level, as shown in Figure 1. The total stress in the two sides of the coal and rock mass within a specific range consists of two parts: (1) the stress caused by the gravity of overlying strata within the outrange of breaking arch and (2) the stress transferred from broken rock beam within the breaking arch of stope. If the total stress is greater than its strength, the coal and rock mass will be broken, and then the peak value of abutment pressure will be transferred inside the coal and rock body [11]. In the breaking process of every rock beam, it forms stress arch that is composed of abutment pressure peak value of every rock strata outside of the breaking arch. Its shape is parabolic in the vertical plane, as shown in Figure 1.

### 2.1. Characteristics of Mechanical Structure of Breaking Arch and Its Evolution Law

The rock strata in the breaking arch play a leading role on the mining pressure, so the research on the characteristics of mechanical structure of breaking arch has a significant meaning for the structural stability of mining stope.

The mining process of heading face can be divided into two stages: (1) no-full mining stage, which means the advancing length of heading face *L*_*x*_ is less than that of the heading face width *L*_0_ and (2) full mining stage, which means the advancing length of heading face *L*_*x*_ is greater than that of the heading face width *L*_0_.

In the no-full mining stage, the height of the overlying spatial structure of stope is linear to the advancing distance of the heading face, which develops forward along the coal mine strike and upward in the space. The height is about half of the short side width of goaf. But this development law is limited. From the analysis above, the height of the overlying strata spatial structure of stope is mainly determined by the width of heading face, which means the maximum height of the overlying strata spatial structure of stope is constant when the width of heading face is fixed. When the advancing length of heading face is less than that of the heading face width, the height of the stope structure is relevant to the advancing length. When the advancing length is greater than the heading face width, the height of the stope structure is half of the heading faces width.

### 2.2. Mechanical Characteristics of Stress Arch and Its Evolution Law

The rock stratum in the stress arch is the main bearing body, which bear and transfer the load of overlying rock strata. The breaking arch structure is located in the distressed zone of the stress arch [12, 13]. When the structure of overlying rock strata in the stress arch fails, the rock burst may occur. So clearly the dynamic evolution process of stress arch must be done.

**Figure 2:**
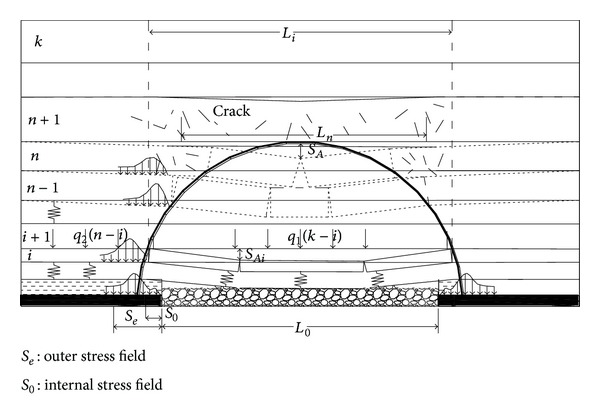
The stress model of out-arch strata.

Assuming the total amount of rock strata is *K* in the overlying rock structure of stope, the *n* + 1 rock stratum which lies on the breaking arch is called supporting stratum. When the *i*th rock stratum breaks, the strata load, originally supported by the *i*th rock stratum, is transferred to the outside of the breaking arch, as shown in Figure 2. So the load, supported by the unit length of *i*th rock stratum outside of the breaking arch, is

(1) $$\begin{array}{c} q_{i}=q_{1(k-i)}+q_{2(k-i)}=\gamma H_{i}L_{i}+\gamma H_{i}=\gamma H_{i}\left(1+L_{i}\right), \end{array}$$

where *q*_1(*k*−*i*)_ and *q*_2(*k*−*i*)_ are the loads of *i*th rock stratum inner and outer field of breaking arch, respectively, *H*_*i*_ is the depth of *i*th rock beam, and *L*_*i*_ is the breaking length of *i*th rock beam.

**Figure 3:**
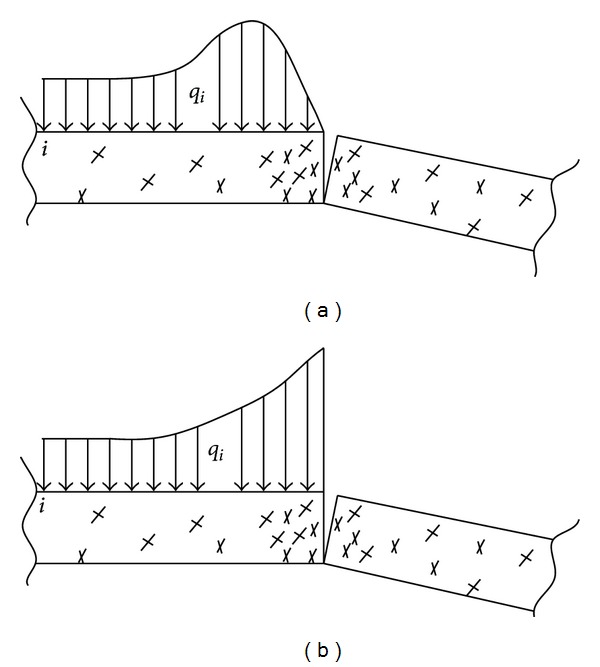
The abutment stress distribution of strata.

The tensile failure of rock beam occurs; lots of tensile fracture may appear in the breaking edge and the load increases quickly, which decrease the strength of rock beam at the breaking position and transfer the abutment pressure peak outside of the breaking arch, as shown in Figure 3(a). It usually happens in the deep coal mines. If the strength of the rock beam is big enough to support the load transferred from the breaking arch with no damage, the abutment pressure peak is at the breaking position, as shown in Figure 3(b). It usually happens in the shallow coal mines.

The width of the stress arch is *L*_stress_ = *L*_0_ + 2*S*_*e*_. The height of the stress arch is *H*_stress_ = *L*_0_/2 + *H*_*n*+1_.

## 3. Mechanical Characteristics of Coal Body Destruction

**Figure 4:**
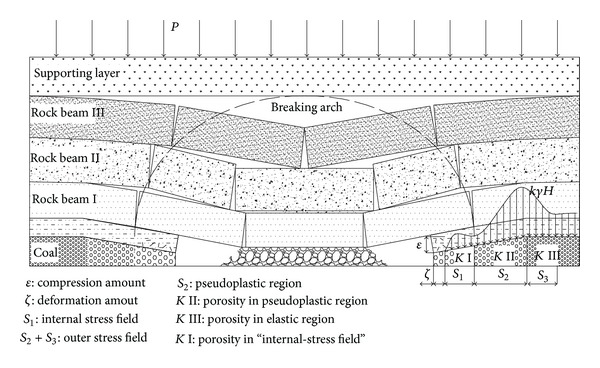
The mechanical model with different time of mining stope.

On the microlevel, the coal body has lots of randomly distributed defects (e.g., pores and microcracks). With the increase of the abutment pressure, the coal body has cumulative damage. If the abutment pressure is greater than the yielding strength of the coal body, the defects propagate quickly; the coal body will become block shaped, as shown in Figure 4.

The broken coal body has irregular shape, so it is really hard to model the breaking process of coal body through mathematical method [14, 15]. Fractal method provides an idea to solve fractal dynamic behavior of geotechnical material by using fractal theory [16]. But the mechanical mechanisms of geotechnical material deformation and failure are not set up, and the relationship between fractal evolution and its physical and mechanics parameters is not built up.

### 3.1. Mechanical Parameters of Macroscopic Coal Body

#### 3.1.1. Mechanical Parameter of the External Stress Field

**Figure 5:**
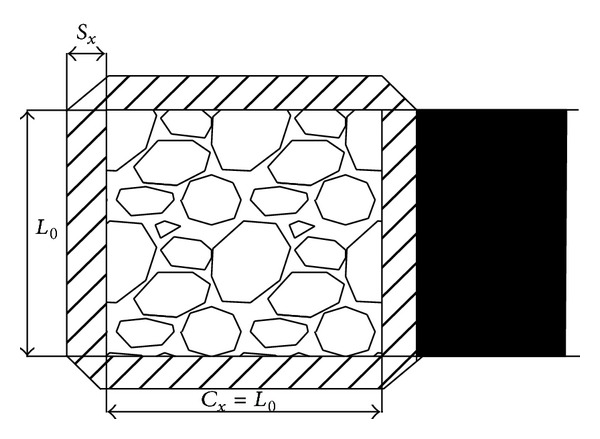
Abutment pressure distribution in the stope.

It should be noted that the abutment pressure range in front of the stope coal wall is up to its maximum when the advancing distance of heading face equals to the width of heading face. With the advance of heading face, the range is basically constant. Its model is shown in Figure 5.

When the advancing distance reaches the width of heading face, the overlying rock strata form an increasing pressure zone with the width of *S*_*x*_ due to its own gravity. Without considering the gangue bearing capacity of goaf, the following expression is built up:

(2) (2L0Sx+2CxSx+2Sx2)(Ka−1)γH  =L0CxγH−12π(12L0)(Hg)γgCx,

where *K*_*a*_ is the average value of stress concentration factor; *H*_*g*_ is the height of stope stress arch with the unit of m; *C*_*x*_ is equal to mining face width that is *L*_0_.

Then,

(3) Sx=−2L0±4L02+2((L02−(1/4)πL02Hg)/(ka−1)H)2.

Simplification,

(4) $$\begin{array}{c} S_{x}\approx \left(\sqrt{1+\frac{4H-\pi H_{g}}{8\left(k_{a}-1\right)H}}-1\right)L_{0}. \end{array}$$

#### 3.1.2. Mechanical Parameters of the Internal Stress Field

1. Stress Intensity.

Based on the assumption that the vertical abutment pressure distributed within the internal stress field around the goaf equals the gravity of the basic roof strata prior to the coming of the initial pressure on the heading face, after formation of the breaking arch, the dynamic structural mechanical model of stope becomes steady, and the distribution range of internal and external stress fields remains stable. As follows in Figure 5, the internal stress field is given by

(5) $$\begin{array}{c} \frac{\sigma_{y\max}}{K_{\max}\gamma H}=\frac{S_{0}/2}{S_{1}}. \end{array}$$

Then,

(6) $$\begin{array}{c} \sigma_{y\max}=\frac{S_{0}K_{\max}\gamma H}{2S_{1}}, \end{array}$$

where *S*_1_ is the distance between peak abutment pressure location and coal wall with the unit of m, *S*_0_ is the range of the internal stress field with the unit of m, *H* is the mining depth with the unit of m, *γ* is the stratum unit weight of kN/m^3^, and *K*_max⁡_ is the stress concentration factor.

(2) Distribution Range.

**Figure 6:**
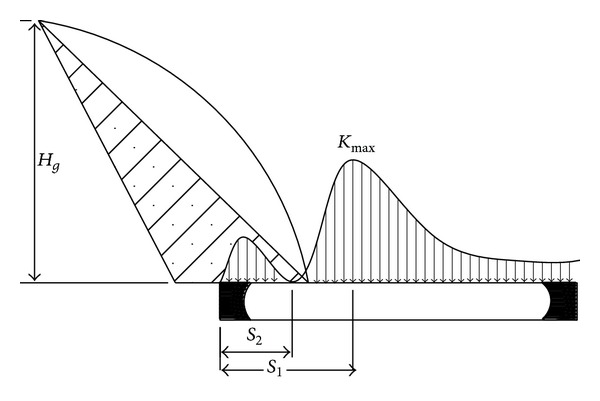
Pressure bearing model.

As can be seen in Figure 6,

(7) $$\begin{array}{c} \frac{1}{2}\sigma_{y\max}S_{0}=\frac{1}{2}\frac{H_{g}C_{i}\gamma }{2}, \end{array}$$

where *C*_*i*_ is the cycle stress step of basic roof strata with the unit of m and *h*_*i*_ is the strata thickness of *i*th layer with the unit of m.

Substituting (7) into the above equation, the range of the internal stress field is given by

(8) $$\begin{array}{c} S_{0}=\sqrt{\frac{2C_{i}H_{g}S_{1}}{K_{\max}H}}. \end{array}$$

### 3.2. Mechanical Parameters of Microscopic Coal Body

Coal body damages under the condition of abutment pressure and its structural forms are very complicated and have different size. The quantitative analysis for the distribution characteristics of coal blocks is essential for the development of abutment pressure. But the stope movement and the development of abutment pressure is a dynamic process, which makes the development and change of pore size have the characteristics of space-time effect during the dynamic process. So the reasonable determination of the relationship between the pore size of the coal body and the dynamic movement of overlying rock strata is helpful to build up the transition between the microscopic quantity and macromechanics parameters.

In the breaking process of rock body, it produces lots of rock blocks with different size, but these blocks are characterized by self-similarity. Assuming the pore size of a rock is *r*, the distribution function is *F*(*r*, ) when the compressed volume of coal wall is *ε*; namely, accumulative percentage of pore size of *r* is

(9) $$\begin{array}{c} F\left(r,\varepsilon \right)=\frac{N\left(r\right)}{N_{0}}, \end{array}$$

where *N*(*r*) is the amount of pores when the compressed volume of coal wall is *ε* and *N*_0_ is the initial amount of the pores.

**Figure 7:**
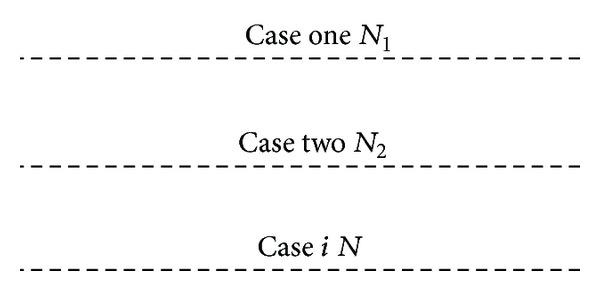
The particle sizes of Void.

For the pores in the internal stress field, assuming segments stand for pore size, according to the length of segments, they can be divided into *n* rows [17]. The *i*th row has the amount of pores *N*_*i*_(*i* = 1,2, 3,…, *n*), as shown in Figure 7.

In Figure 7, ∑_*i*=1_^*n*^ *N*_*i*_ = *N*_0_; then,

(10) $$\begin{array}{c} N\left(x\right)=\frac{N_{0}}{{\left(r/r_{\max}\right)}^{-D}},\; \end{array}$$

where *N*_0_ is constant and *D* is the fractal dimension of pore size.

Substituting (10) into (9), in accordance with *F*(*r*_max⁡_) = 1, the expression for the distribution fractal dimension of pore size of coal body under different compressed volume is given by

(11) $$\begin{array}{c} F\left(r,\varepsilon \right)={\left(r/r_{\max}\right)}^{-D}, \end{array}$$

where *r* is accumulative percentage of pore size and *D* is the fractal dimension of pore size.

### 3.3. Analysis of Extension Mechanism of Internal Stress Field

On the microlevel, last section analyses the outburst mechanism of coal wall, but the parameters are hard to obtain. Based on the field application, this paper provides a brief analysis of the extension mechanism of internal stress field from the macroperspectives.

**Figure 8:**
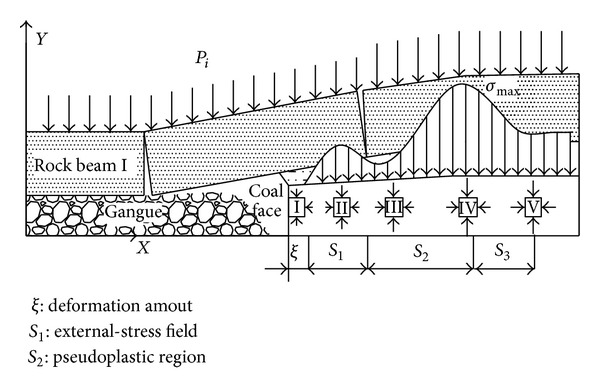
The mechanical model with coal body being loaded.

As can be seen from Figure 8, the stress states of coal body at the coal wall are biaxial compressive stress, and the external coal body is triaxial compressive stress.

Assuming that the porosity of coal body is *n*, the vertical stress is *σ*, then the lateral pressure is *p* = *f*(*n*, *σ*). The vertical stress *σ* is basically constant, so the change of lateral pressure can be analyzed by using the porosity change indirectly.

**Figure 9:**
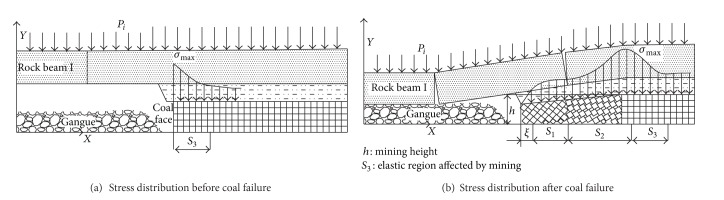
The coal body failure model.

As can be seen in Figure 9, the volume of coal body within the unit length of internal stress field is *V*_0_ = *hS*_1_; after compression, the volume becomes

(12) $$\begin{array}{c} V_{1}=\frac{\left(2h-\varepsilon \right)\left(S_{1}+\xi \right)}{2}.\;\; \end{array}$$

Then the volume difference between the uncompressed volume and compressed volume is given by

(13) ΔV=V1−V0=hξ−ε2(S1−ξ),         ε=ξtanθ.  

Then,

(14) $$\begin{array}{c} \Delta V=\xi \left(h-\frac{S_{1}}{2}\tan\theta \right).\; \end{array}$$

1. If *h* − (*S*_1_/2)tan*θ* > 0, then Δ*V* > 0. The volume of coal body increases due to the increase of the porosity. At this moment, the lateral stress decreases at the interface between the external and the internal of stress field and the coal body becomes imbalance, which extends the range of internal stress field.
2. If *h* − (*S*_1_/2)tan*θ* < 0, then Δ*V* < 0. The volume of coal body decreases due to the compression reducing the porosity. At this moment, the lateral stress increases at the interface between the external and the internal of stress field and the coal body becomes balance, so the range of internal stress field is basically constant.
3. If *h* − (*S*_1_/2)tan*θ* = 0, then Δ*V* = 0. The volume and the porosity of coal body do not change, so the range of internal stress field is basically constant.

### 3.4. Solution of the Range of Coal Deformation

The Sierpinski gasket, which has the characteristics of self-similarity, is used to analyse the fractal particle size distribution of real unit system.

**Figure 10:**
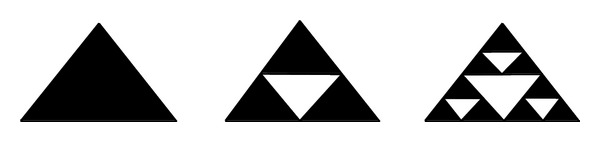
The Sierpinski gasket formation.

In Figure 10, the initial model is an equilateral triangle. Then it is cut into quarters, removing the middle one and keeping the three sides; this is called one generation [18, 19]. Cutting the other three small equilateral triangles into quarters, with removing the middle parts and keeping their sides, is called the second generation. Repeating the operation until infinity, the Sierpinski gasket will be done.

Assuming that the gasket size is limited, the pore size is also limited. The pore size in the *k*th generation is *L*_*k*_, (*k* = 0,1, 2,…) with the maximum size of *L*_*c*_ and minimum size of *L*_*f*_. *N* stands for the amount of pores whose size is bigger than *L*_*k*_. For the initial model, *L*_0_ = *L*_*c*_. So the relationship between the amount of pores and the pore size is shown in Table 1.

From Table 1, the amount of pores *N* is given by

(15) $$\begin{array}{c} N=\frac{1}{2}\left(3^{k}-1\right),\;\\ 3^{k}=2^{k(\ln3/\ln2)}=2^{kD}=L_{k}^{-D}L_{c}^{D}.\;\; \end{array}$$

Then,

(16) $$\begin{array}{c} N=\frac{1}{2}L_{c}^{D}\left(L_{k}^{-D}-L_{c}^{-D}\right).\; \end{array}$$

Equation (16) is the expression for fractal distribution of pore during the breaking process of coal body.

In order to qualitatively analyse the mechanical feature during the compressive process of coal body, we assume that the elements and pore body are generated by using the Sierpinski gasket. The area of initial element is 1, and the relationship between the amount and the size is shown in Table 2.

The area of pore body of coal body is given by

(17) $$\begin{array}{c} S_{0}=\frac{3^{k}}{2_{2k}},\;\left(k=1,2,3,\ldots \right). \end{array}$$

Then, the total volume of pore in coal body is given by

(18) $$\begin{array}{c} V_{\text{pore}}=NS_{0}=\left(1-{\left(\frac{3}{4}\right)}^{k}\right),\;\left(k=1,2,3,\ldots \right).\;\; \end{array}$$

With the assumption of the area of deformed coal body of *V*_coal_ = *S*_1_ *h*, the range of coal outburst is given by

(19) $$\begin{array}{c} \xi =S_{1}\left[1-{\left(\frac{3}{4}\right)}^{k}\right],\;\left(k=1,2,3,\ldots \right), \end{array}$$

where *S*_1_ is the width of the deformed coal body with the unit of m and *h* is the height of the deformed coal body with the unit of m.

## 4. Conclusion

1. The spatial form of stope model can be divided into two parts: the outrange of overlying strata spatial structure of stope that consists of no significant move rock beams in the stress arch that does not move significantly and the overlying strata spatial structure that is composed of kinetic rock strata in the breaking arch that affects the ground pressure of stope directly.
2. According to mechanical balance model of stope, it solves the mechanical parameters of stress field and modifies the expression for the distribution range of internal and external stress field. The range of internal stress field is 1.4 times greater than that of itself calculated by the traditional method.
3. The solution for the distribution range of abutment pressure can be divided into two stages. When the advancing length of heading face is less than that of the heading face width, the width is expressed by $S_{x}\approx (\sqrt{1+\left(\left(4H-\pi H_{g}\right)/8(k_{a}-1)H\right)}-1)L_{0}$. When the advancing length of heading face is greater than that of the heading face width, the width is *S*_*x*max⁡_ ≤ *L*_0_(4*H* − *πH*_*g*_)/8(*k*_*a*′_ − 1)*H*. The expression for the range of internal stress field is $S_{0}=\sqrt{2C_{i}H_{g}S_{1}/K_{\max}H}$.
4. The broken coal body has the characteristic of self-similarity. This paper studies the relationship between block degree of coal body and damage variables and extension mechanism of internal stress field. According to the Sierpinski gasket method, the pore area in coal body is given by *V*_pore_ = *NS*_0_ = (1 − (3/4)^*k*^) and the range of coal outburst is given by *ξ* = *S*_1_[1 − (3/4)^*k*^], (*k* = 1,2, 3,…).

## Figures and Tables

**Table 1:** The void distribution of Sierpinski gasket.

*k*	0	1	2	3	4	⋯	*k*	⋯
*L*_*k*_	*L*_*c*_	2^−1^ *L*_*c*_	2^−2^ *L*_*c*_	2^−3^ *L*_*c*_	2^−4^ *L*_*c*_	⋯	2^−*k*^ *L*_*c*_	⋯
*N*	0	1	4	13	40	⋯	(3^*k*^ − 1)/2	⋯

**Table 2:** The area distribution of Sierpinski gasket.

*k*	0	1	2	3	4	⋯	*k*	⋯
*L*_*k*_	*L*_*c*_	2^−1^ *L*_*c*_	2^−2^ *L*_*c*_	2^−3^ *L*_*c*_	2^−4^ *L*_*c*_	⋯	2^−*k*^ *L*_*c*_	⋯
*S*_pore_	0	1/4	7/16	37/64	91/256	⋯	1 − (3/4)^*k*^	⋯

## References

[1] Miao XX, Qian MG (2003). Broken feature of key strata and its influence on rock pressure in super-length fully-mechanized coal face. *Chinese Journal of Rock Mechanics and Engineering*.

[2] Jiang FX, Zhang XM, Yang SH, Xun L, Ma Q, Wang H (2006). Discussion on overlying strata spatial structures of longwall in coal mine. *Chinese Journal of Rock Mechanics and Engineering*.

[3] Kanamori H, Kikuchi M (1993). The 1992 Nicaragua earthquake: a slow tsunami earthquake associated with subducted sediments. *Nature*.

[4] Mitra R, Westman EC (2009). Investigation of the stress imaging in rock samples using numerical modeling and laboratory tomography. *International Journal of Geotechnical Engineering*.

[5] Tan YL, Zhao TB, Xiao YX (2010). Researches on floor stratum fracturing induced by antiprocedure mining underneath close-distance goaf. *Journal of Mining Science*.

[6] Hou CJ, Li XH (2001). Stability principle of big and small structures of rock surrounding roadway driven along goaf in fully mechanized top coal caving face. *Journal of China Coal Society*.

[7] Zhang G, Du Y, Zhang Y (2013). Analyses and the study of syngas production in dual-gas resources polygene ration. *Asian Journal of Chemistry*.

[8] Jiang FX (2006). Viewpoint of spatial structures of overlying strata and its application in coal mine. *Journal of Mining & Safety Engineering*.

[9] Yang K, Xie GX (2007). Stress field of surrounding rocks of fully mechanized top-coal caving gateway with small pillars. *Journal of Mining Safety Engineering*.

[10] Song ZQ (1988). *Practical Ground Pressure Control*.

[11] Hasson A, Jweeg M (2013). Soil organic carbon sequestration under pastures in arid region. *Nature Environment & Pollution Technology*.

[12] Zhang G, Du Y, Zhang Y, Xu Y (2014). Desulfurization reaction model and experimental analysis of high sulfur coal under hydrogen atmosphere. *Journal of Industrial and Engineering Chemistry*.

[13] Sheng JL, Zhu RG (2000). The fractal evaluation of joint roughness coefficient. *Journal of Wuhan University of Science and Technology (Natural Science Edition)*.

[14] Mandelbrot BB (1982). *The Fractal Geometry of Nature*.

[15] Qin Huang QH, Angelier J (1989). Fracture spacing and its relation to bed thickness. *Geological Magazine*.

[16] Ladeira FL, Price NJ (1981). Relationship between fracture spacing and bed thickness. *Journal of Structural Geology*.

[17] Yu BM (2001). Sorne fraetal charaeters of porous media. *Fractals*.

[18] Xiao J-W, Yi Y (2007). Coupled-adaptive synchronization for Chen chaotic systems with different parameters. *Chaos, Solitons and Fractals*.

[19] Procházka PP (2004). Application of discrete element methods to fracture mechanics of rock bursts. *Engineering Fracture Mechanics*.

